# Assessing quality of life in older adults: psychometric properties of the OPQoL-brief questionnaire in a nursing home population

**DOI:** 10.1186/s12955-019-1245-3

**Published:** 2020-01-02

**Authors:** Gørill Haugan, Jorunn Drageset, Beate André, Kamile Kukulu, James Mugisha, Britt Karin S. Utvær

**Affiliations:** 1 NTNU Center for health promotion research, Norwegian University of Science and Technology, Trondheim, Norway; 2 Faculty of Nursing and Health Sciences, Nord University, Bodø, Norway; 3 Faculty of Health and Social Science, Western University of Applied Science, Bergen, Norway; 4 University of Bergen, Bergen, Norway; 5 Faculty of Nursing, Akdeniz University, Antalya, Turkey; 6 Social Worker, Kyambogo University and Butabika National Referral and Teaching Hospital, Kampala, Uganda; 7 Department of Teacher Education, Norwegian University of Science and Technology, Trondheim, Norway

**Keywords:** Factor analysis, Nursing home residents, Nursing home care, OPQoL-brief questionnaire, Psychometric properties, Quality of life, Wellbeing

## Abstract

**Background:**

Well-adapted and validated quality-of-life measurement models for the nursing home population are scarce. Therefore, the aim of this study was to test the psychometrical properties of the OPQoL-brief questionnaire among cognitively intact nursing home residents. The research question addressed evidence related to the dimensionality, reliability and construct validity, all of which considered interrelated measurement properties.

**Methods:**

Cross-sectional data were collected during 2017–2018, in 27 nursing homes representing four different Norwegian municipalities, located in Western and Mid-Norway. The total sample comprised 188 of 204 (92% response rate) long-term nursing home residents who met the inclusion criteria: (1) municipality authority’s decision of long-term nursing home care; (2) residential time 3 months or longer; (3) informed consent competency recognized by responsible doctor and nurse; and (4) capable of being interviewed.

**Results:**

Principal component analysis and confirmative factor analyses indicated a unidimensional solution. Five of the original 13 items showed low reliability and validity; excluding these items revealed a good model fit for the one-dimensional 8-items measurement model, showing good internal consistency and validity for these 8 items.

**Conclusion:**

Five out of the 13 original items were not high-quality indicators of quality-of-life showing low reliability and validity in this nursing home population. Significant factor loadings, goodness-of-fit indices and significant correlations in the expected directions with the selected constructs (anxiety, depression, self-transcendence, meaning-in-life, nurse-patient interaction, and joy-of-life) supported the psychometric properties of the OPQoL-brief questionnaire. Exploring the essence of quality-of-life when residing in a nursing home is highly warranted, followed by development and validation of new tools assessing quality-of-life in this population. Such knowledge and well-adapted scales for the nursing home population are beneficial and important for the further development of care quality in nursing homes, and consequently for quality-of-life and wellbeing in this population.

## Background

Currently, the world faces a shift to an older population;125 million people are now aged 80 years or older [1]. While this shift started in high-income countries (for example in Japan 30% of the population are already over 60 years old), it is now low- and middle-income countries that are experiencing the greatest change. Today, most people can expect to live into their sixties and beyond [1]. Between 2015 and 2050, the proportion of the world’s population over 60 years will nearly double from 12 to 22%; by 2050, the world’s population aged 60 years and older is expected to total 2 billion, up from 900 million in 2015 [1, 2]. All countries in the world face major challenges to ensure that their health and social systems are ready to make the most of this demographic shift [1].

As people live longer it is important to ensure that the extra years of life are worth living, despite chronic illnesses. Quality-of-life (QoL) and health promotive initiatives for older persons living in nursing homes (NH) will become ever more important in the years to come. The World Health Organization Quality of Life Group [3] defined QoL as an “individuals’ perception of their position in life in the context of the culture and value systems in which they live and in relation to their goals, expectations, standards and concerns.” Additionally, global QoL encompasses multiple constructs such as physical health, psychological status, independence level, social relationships, and relationship with significant features of the environment [3].

QoL conceptual models and instruments for research, evaluation and assessment in diverse populations have been developed since the middle of last century [4, 5]. However, well-adapted and validated QoL measurement models for the NH population are scarce. Accordingly, our understanding of QoL among individuals living in NHs is still limited. During the last decades, spirituality [6–8], a sense of meaning-in-life, hope, self-transcendence [9–17], social relationships and social support [5] have become vital aspects of wellbeing and QoL among older adults.

Global QoL is a multifaceted concept, representing a subjective state which is affected by chronic and debilitating health conditions [18]; such conditions are common among older adults in NHs. Research shows that the NH population is characterized by high age, frailty, mortality, disability, powerlessness, dependency, vulnerability, poor general health and a high symptom burden [19–21]. Accordingly, moving to a NH results from numerous losses, illnesses, disabilities, loss of functions and social relations, and facing the end-of-life, all of which detrimental to people’s functioning, independency and QoL. Moreover, older people experience changes in roles, relationships, and living environments that can increase their risk for experiencing social isolation and loneliness [5]; particularly when moving to a NH. With advancing age, it is inevitable that people lose connection with their friendship networks and that they find it more difficult to initiate new friendships and to belong to new networks. However, a link between QoL and connectedness is emerging in the literature [5]. Despite old age, chronical diseases or frailty; the desire for affiliation and social bonding is an intrinsic human need, also when living in a NH. Hence, the life situation for older adults in NHs might differ significantly from other older adult populations, staying at home or in hospitals. Consequently, a valid and reliable scale assessing QoL in this population is important for the further development of care quality and health promoting intervention in NHs.

While planning for the present study, we searched, broadly and thoroughly, for a valid and reliable measure of QoL suitable for the NH population. Along this road, we found the OPQoL-brief questionnaire [22], which was developed by a “bottom-up” approach and tested among older adults in Britain [22]. In a frail and vulnerable population such as the NH population, a shorter scale is warranted. Though shorter instruments are more limited in scope and sensitivity than longer measures, the benefits are reduced respondent and research burden and costs. The OPQoL-brief is a shortened version of the OPQOL-35 showing good psychometrics among older adults [20]; therefore, the OPQoL-brief was selected for this Norwegian study. To the authors knowledge, the OPQoL-brief has not previously been tested by means of confirmatory factor analysis as well as among NH residents.

### Aims

The aim of this study was to assess the psychometric properties of the Norwegian version of the OPQoL-brief questionnaire in a cognitively intact (not diagnosed with dementia and recognized by the responsible doctor and nurse to have informed consent competency) NH population. The research question was two-fold; (a) how well does the original one-factor measurement model of the OPQoL-brief fit to the observed data? (b) Does the OPQoL-brief reveal good reliability and construct validity in a NH population? We expected the OPQoL- brief to correlate with some established concepts, and tested the following hypotheses:

- Hypotheses1 (H1): OPQoL-brief correlates negatively with anxiety and depression.
- Hypotheses2 (H2): OPQoL-brief correlates positively with self-transcendence, meaning-in-life, nurse-patient interaction and joy-of-life.

In accordance to the Standards for Educational and Psychological Testing [23, 24], the present research question addressed evidence related to the dimensionality, reliability and construct validity, all of which considered interrelated measurement properties. *Dimensionality* examines the extent to which the internal components of a test match the defined constructs, and is concerned with the homogeneity of the items [25]. *Reliability* involves an instrument’s consistence and relative lack of error [25]. This study assessed internal consistence by the reliability coefficients Cronbach’s alpha (α) and composite reliability (ρ_c_). *Construct validity* refers to how well a scale actually measures the construct it is intended to measure, and is based among others on the constructs’ relationships to other variables [25]. There are two subsets of construct validity: convergent construct validity and discriminant construct validity. Convergent construct validity tests the relationship between the construct and a similar measure; this shows that constructs which are meant to be related are related. Discriminant construct validity tests the relationships between the construct and an unrelated measure; this shows that the constructs are not related to something unexpected. In order to have good construct validity one must have a strong relationship with convergent construct validity and no relationship for discriminant construct validity [26]. In line with the WHO statement of health, salutogenic concepts such as meaning, self-transcendence, joy-of-life and nurse-patient-interaction are found to enhance NH residents’ QoL [10, 17, 20, 27–35], and to decrease anxiety and depression [36–38]. Therefore, these constructs were selected for assessing convergent construct validity by means of correlational analyses.

*Content validity* refers to the degree to which a scale has an appropriate, relevant sample of items to represent the construct of interest—that is, whether the content of the specific construct is adequately represented by the items, meaning that the indicators measure all ideas in the theoretical definition [39]. A frequent challenge occurs when the wording of items is too similar—namely, the coefficient alpha, as well as the content validity and dimensionality, are artificially enhanced. Nevertheless, items worded too similarly increase the average correlation among items, which in effect increases the coefficient alpha, yet without adding substantively to the content validity of the measure. Although some similarity among items of a scale is needed to tap into the domain, several items that are mere rewordings of other items are redundant and contain very little new information about the construct [40]. In that sense, theory, validity, reliability, and dimensionality are intertwined.

## Methods

### Design and data collection

Data were collected during 2017–2018 in 27 NHs representing two small and one large urban municipality in Mid-Norway and a large urban municipality in Western Norway. The total sample comprised 188 of 204 (92% response rate) long-term NH residents who met the inclusion criteria: (1) municipality authority’s decision of long-term NH care; (2) residential time 3 months or longer; (3) informed consent competency recognized by responsible doctor and nurse; and (4) capable of taking adequately part in an interview situation. A nurse at the actual ward presented potential participants with oral and written information about the study, their rights as participants and their right to withdraw at any time.

Due to impaired vision, problems holding a pen, fatigue etc., this population have difficulties completing a questionnaire on their own. Therefore, six trained researchers (3 in each part of Norway) conducted one-on-one interviews in the resident’s private room in the NH. Researchers with identical professional background (RN, MSc, trained and experienced in communication with elderly, as well as teaching gerontology at an advanced level) were trained to conduct the interviews in the same manner. The OPQoL-brief was part of a battery of seven scales comprising in total 120 items. To avoid misunderstandings, interviewers read each question loudly, and held a large-print copy of questions and possible responses in front of the participants.

### Participants

Participants ages ranged between 63 and 104 years (mean 87.4 years, SD = 8.6). The sample consisted of 132 women (73.3%) and 48 men (26.7%), where the mean age for women was 88.3 years (SD = 1.8) and 86 years (SD = 1.2) for the men. In total, 23 were married, 22 cohabitating, 1 was single, 106 were widows/widowers, and 37 were divorced.

### Instruments

The measure of QoL analyzed here is the OPQOL-brief – the short form of the OPQOL-35 questionnaire which was designed to assess QoL among older adults 65+ [41, 42]. The OPQOL-35 has been validated on community-dwelling older populations, and ethnically diverse population samples in Britain [43, 44]. The OPQOL-35 was further tested among geriatric service out-patients in Italy showing excellent applicability to cognitively intact older people, and also to be applicable to most of the people suffering from mild or moderate dementia [45–47]. The OPQOL-35 assumes that QoL is a multidimensional concept; the original version includes eight domains [43, 44]. Nevertheless, the factor structure has shown to be unclear; studies of the OPQOL-35 have reported two [43], four [43], seven [48], or nine-factor solutions [42] based on principal component analysis (PCA). Like the original 35-items version [43], Chen [49] extracted eight factors using PCA. No other factor analyses are currently available. A more detailed examination of the factor structure by means of CFA is needed [48].

*The OPQoL-brief* comprises of 13 items which are scored Strongly agree = 1, Agree = 2, Neither = 3, Disagree = 4, Strongly disagree = 5 [22]. The items are summed for a total OPQoL-brief score, then positive items are reverse coded, so that higher scores represent higher QoL. The total sum-score ranges from 13 to 65. Examples of items include enjoying one’s life, looking forward to things, staying involved with things, and feeling safe where one lives, etc. (Table 4 in Appendix 1). The OPQoL-brief was found to be a highly reliable and valid measure of QoL in old age [22]. For use in this study, two experts of both languages, English and Norwegian, translated the OPQoL-brief into Norwegian, following the procedure of back-and-forth translation. To better reflect the nuances of the target language [50], two independent translators did the forward translation into Norwegian (their mother tongue) [51]. One of these was a naive translator who was unaware of the objective of the questionnaire, while the other was a researcher in the field of QoL. No discrepancies appeared. To assure the accuracy of the translation, the initial translation was independently back-translated (from Norwegian into English) by two independent translators. The back-translators were not aware of the intended concept the questionnaire was [52].

*The Self-Transcendence Scale (STS)* [53] assessed interpersonal and intrapersonal self-transcendence. The STS comprises 15 items, each with a score of 1–4, reflecting expanded boundaries of self which are considered to be characteristics of a matured view of life [54]. Total score ranges between 15 and 60, where higher scores indicate higher ST. The STS has shown good psychometric properties [55, 56] and has been translated into Norwegian, and validated in NH patients [56] showing a two-factor-construct (STS1 & STS2) to be most valid and reliable [54]. The present study applied this two-factor construct (ST1, ST2).

*The Purpose-in-Life Test (PIL)* assessed meaning-in-life. Based on Frankl’s theory, the PIL was designed to be a general tool assessing meaning [57–60] and has been commonly used for this purpose [61–63]. The PIL is translated into Norwegian [64] and has previously been used with elderly individuals up to 104 years old [65–67]. The Norwegian version has been validated among NH residents, showing good psychometric properties [66]. Each statement is scored from 1 to 7; four represents a neutral value, whereas the numbers from 1 to 7 stretch along a continuum from one extreme feeling to the opposite kind of feeling; higher scores reflect higher meaning-in-life [60]. Total score ranges from 20 to 140.

*The Joy-of-Life scale (JoLS)* was developed in Norway to assess NH patients’ perceived joy-of-life (JoL) [68]. The intention was to identify essential characteristics of NH patients’ experiences of JoL in their daily life. The JoLS covers domains that identify fundamental qualities stressed in well-being theory [69–72], nursing care literature [73–76], and the dimensions found by 29 in-depth interviews on the essence of joy-of-life with NH residents [77]. A 13-items version of the JoLS was found reliable and valid in this population (Haugan, Rinnan et al.2019), and was applied in the present study.

*The Nurse-Patient-Interaction Scale* (NPIS) developed in Norway, assessed perceived nurse-patient-interaction. The NPIS comprises 14 items identifying essential relational qualities stressed in the nursing literature [31]. This scale is scored from 1 (not at all) to 10 (very much); total score ranges between 14 and 140, where higher numbers indicate better perceived nurse-patient-interaction. The NPIS has shown good psychometric properties with good content validity and reliability among NH residents [31].

*The Hospital Anxiety and Depression Scale* (HADS), comprising 14 items, with subscales for anxiety (HADS-A, 7 items) and depression (HADS-D, 7 items) assessed anxiety and depression. Each item is rated from 0 to 3, giving a range of total score between 0 and 21; higher scores indicate more anxiety and depression. The HADS has shown good to acceptable reliability and validity in the NH population [78].

#### Ethical considerations

We obtained approval by the Regional Committee for Medical and Health Research Ethics in Norway (ref.nr 2014/2000/REK Central) as well as from the Management Units at the 27 NHs. Each participant provided voluntarily written informed consent.

### Data analysis

The same data were analyzed by descriptive statistics and principal component analysis (PCA) using IBM SPSS version 25, and confirmatory factor analysis (CFA) by means of Stata 15.1 [79]. When evaluating a measurement scale investigating the underlying dimensionality of data and the adequacy of each individual item is central. In these instances, PCA and CFA can provide complementary perspectives on data, giving different pieces of information [25, 80]. The implicit assumption underlying the use of PCA in the present study is the insecurity with respect to the dimensionality of the OPQoL-Brief, which has not been previously tested by means of CFA, neither among NH residents. As previously presented, the OPQoL-brief is a short version of the original OPQoL scale, which has shown 2,4,7,8 and 9 factors. Therefore, a broad perspective on the observed data using PCA followed by the confirmation procedure was used.

Confirmatory factor analysis (CFA) is a sub-model in structural equation modeling that deals specifically with measurement models [81], accounting for random measurement error, and thus derive a more accurate evaluation of the psychometric properties of the scales used. A high loading of an item indicates that there is much in common between the factor and the respective item [82]. Loadings below 0.32 are considered poor, ≥0.45 fair, ≥0.55 good, ≥0.63 very good, and above 0.71 are excellent [82]. Thus, a good rule of thumb for the minimum loading is .32 [83], which equates to approximately 10% overlapping variance with the other items in the factor. A “cross-loading” item loads at .32 or higher on two or more factors.

The present study assessed model fit adequacy by χ^2^-statistics and various fit indices. In line with the ‘rules of thumb’ given as conventional cut-off criteria [84] the following fit indices were used; χ^2^-statistics, the Root Mean Square Error of Approximation (RMSEA) and the Standardized Root Mean Square Residual (SRMS) with values below 0.05 indicating good fit, whereas values smaller than 0.10 is interpreted as acceptable [85]. Further, the Comparative Fit Index (CFI) and the Tucker-Lewis Index (TLI) with acceptable fit set at 0.90 [84, 86] were used. Both skewness and kurtosis were significant and the Robust Maximum Likelihood (RML) estimate procedure was applied. When analyzing continuous but non-normal endogenous variables, the Satorra-Bentler corrected χ^2^ [87] should be reported [88].

## Results

### Descriptives

**Fig. 1:**
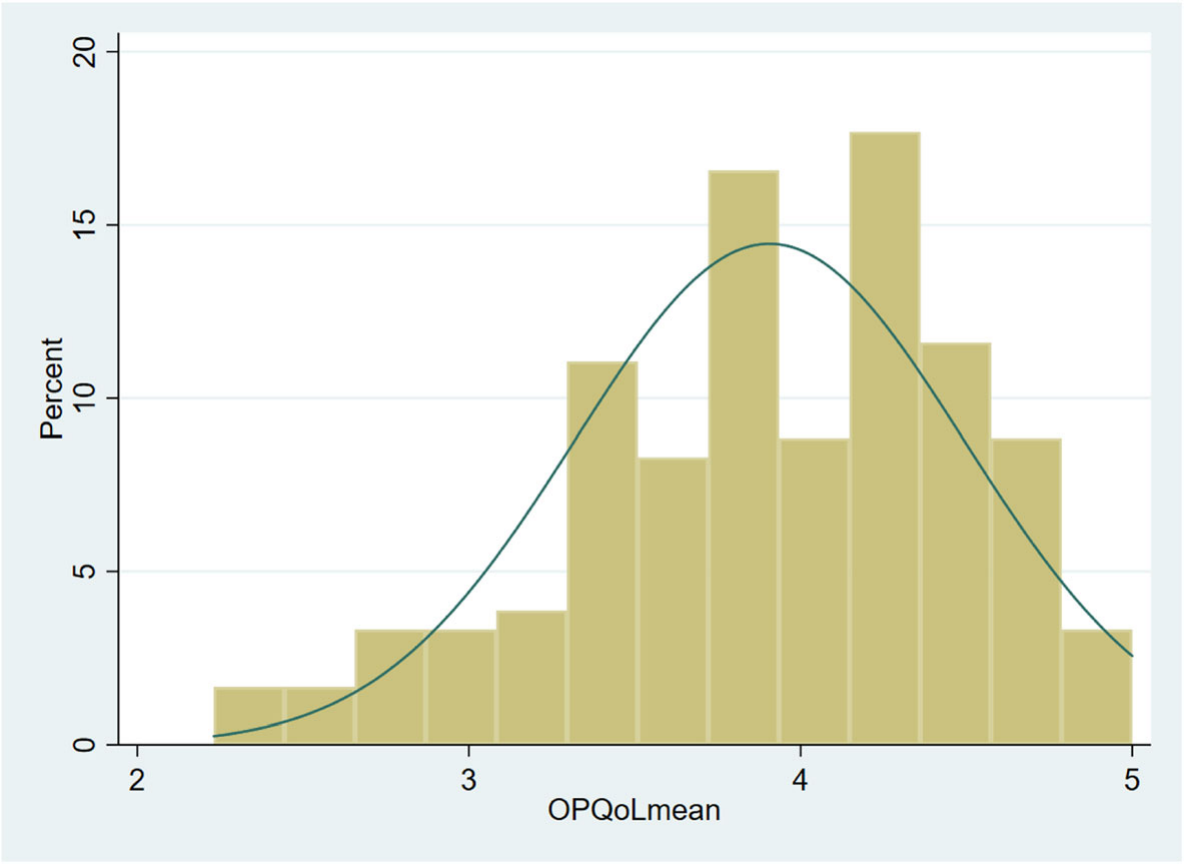
Histogram: The distribution of the OPQoL mean-score, 13-items

The OPQoL-brief 13-items mean-scores ranged between 2.99–4.53, showing a total mean of 3.9 (SD = 1.01). In this study, 11 (6.1%) of the NH residents reported QoL mean-score < 3.0 interpreted as a low QoL, 90 (49.7%) stated a high QoL ≥4.0–5.0, while 77 (42.5%) reported QoL mean-scores between 3.0–3.99, representing a modest QoL. Figure 1 displays the distribution of the OPQoL mean-scores, while Table 1 lists the means, standard deviation, Cronbach’s alpha and correlation matrix for the constructs of QoL, ST1, ST2, PIL, JoL, NPIS, HADS-A and HADS-D.

**Table 1:** Distribution of the OPQoL scores, Means (M), Standard deviations (SD), Cronbach’s alpha, Correlation coefficients for OPQoL to Self-Transcendence, Meaning-in-life, Sense of Coherence, Nurse-patient Interaction, Joy-of-Life, Depression, and Anxiety

Distribution of the OPQoL scores				
OPQoL score 1–2.99 3.0–3.99 4.0–5.0				
*N* = 181 100% 11 (6.1%) 77 (42.5%) 90 (49.7%)				
Variable (number items)	Cronbach’s Alpha (α)	Mean (*M)*	Std.Dev. (*SD*)	*Correlations (r*^*2*^*) OPQoL brief (13)*
OPQoL-brief (13)	0.83	3.901	1.008	1.00
ST1 (7)	0.65	2.518	0.569	0.56**
ST2 (8)	0.68	3.145	0.449	0.47**
PIL (20)	0.80	3.482	1.091	0.40**
NPIS (14)	0.90	7.981	1.907	0.45**
JOL (13)	0.88	5.682	1.137	0.69**
HADS-D (7)	0.74	1.686	0.552	− 0.17**
HADS-A (7)	0.83	1.864	0.410	− 0.29**

*OPQoL-brief* Quality-of-life, *ST1* Interpersonal self-transcendence, *ST2* Intrapersonal self-transcendence, *PIL* Purpose in Life test, *NPIS* Nurse-Patient Interaction, *JoL* Joy-of-Life Scale, *HADS-D* Depression, *HADS-A* Anxiety, N = 181

### Dimensionality

#### Principal Component Analysis (PCA)

**Fig. 2:**
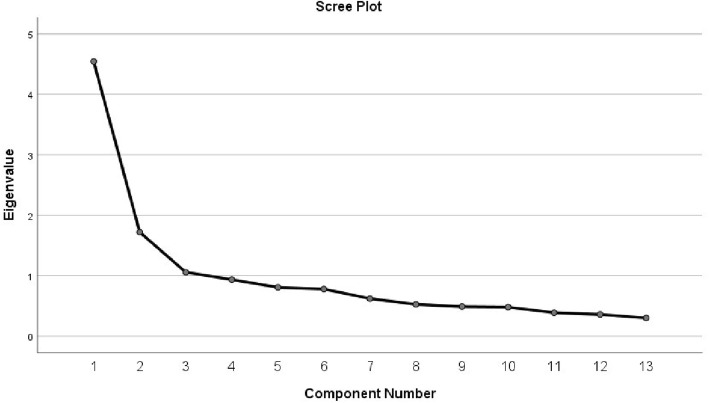
Scree-plot of the OPQoL Brief questionnaire, 13-items

In order to explain as much of the total variance as possible with as few factors as possible, the OPQoL-brief was subjected to PCA. The Kaiser-Meyer-Olkin measure of sampling adequacy exceeded the recommended value of .60 (.84) and Bartlett’s test of sphericity showed statistical significance (*p* < 0.0001), supporting the factorability of the correlation matrix. We search for the cleanest structure of the concept under investigation and expected the OPQoL-brief to be one- or multi-dimensional with correlated factors. Hence, an oblique rotation such as promax should theoretically render a more accurate solution [89]. PCA with promax rotation and Kaiser Normalization were used; three factors with eigenvalue 1.0 and greater (4.82, 1.72 and 1.06, respectively) were extracted (Table 2). Figure 2 portrays the scree-test of the OPQoL-brief data showing the number of factors to retain is three. Table 2 lists the loadings and variance for this rotated 3-factor solution of the OPQoL-brief suggested by PCA. Yet, this 3-factor solution revealed 9 cross-loadings, with substantial factor loadings on all factors indicating an unclear dimensionality.

**Table 2:** Exploratory Factor Analysis of the OPQoL-brief questionnaire – Rotated Component Matrix. Estimates for factor loadings, extraction sums of squared loadings and Cronbach’s alpha

Model-1 (3 factors, 13 items)			
OPQoL1 I enjoy my life overall	.489	.565	.706
OPQoL2 I look forward to things		.713	.632
OPQoL3 I am healthy enough to get out and about	.505	.367	.501
OPQoL4 My family, friends or neighbours will help me if needed		.569	.634
OPQoL5 I have social or leisure activities/hobbies that I enjoy doing		.807	
OPQoL6 I try to stay involved with things		.800	
OPQoL7 I am healthy enough to have my independence	.701		
OPQoL8 I can please myself what I do	.576	.520	.455
OPQoL9 I feel safe where I live	.525		.617
OPQoL10 I get pleasure from my home	.719		.347
OPQoL11 I take life as it comes and make the best of things	.601		.591
OPQoL12 I feel lucky compared to most people	.690		.340
OPQoL13 I have enough money to pay for household bills			.684
Cumulative % of total variance explained	34.967	48.199	56.326
Cronbach’s Alpha *(number of items)*	.75 (6)	.66 (3)	.62 (4)

Extraction Method: Principal Component Analysis. Rotation Method: Promax with Kaiser Normalization. Values< 0.32 are suppressed. Model-1: Three components extracted based on Eigenvalue > 1. Total variance explained: 54,325. Rotation converged in 8 iterations. Listwise *N* = 181

Substantial conclusions based solely on PCA should not be drawn [89]; therefore, we turned to confirmatory factor analysis (CFA).

#### Confirmatory Factor Analysis (CFA)

Firstly, we checked the original 13-items unidimensional version, revealing a very bad fit to the present data. Consequently, we tested the 3-factor solution suggested by the PCA (Factor 1: items 3, 7, 8, 10, 11, 12; Factor 2: items 2, 5, 6, 7 and Factor 3: items 1, 4, 9, 13). Running CFA, this 3-factor-model did not fully converge and did not provide fit indices; both of which indicating misspecifications. The original OPQoL-brief revealed one dimension; and a 3-factor-solution of the OPQoL-brief construct did not seem theoretically meaningful. Therefore, we turned back to the original unidimensional 13-items model [22] for further examination.

### Reliability

#### Model-1 – the original OPQoL-brief unidimensional version

Model-1 comprising 13 items gave significant t-values for all estimates, showing completely standardized factor loadings from .78–.41, and squared multiple correlations (R^2^) ranging between .61–.16. Some items (item4,5,6,13) disclosed low R^2^-values (≤0.19) indicating low reliability. The model fit was bad: χ^2^ = 236.36, (df = 65), χ^2^/df = 3.64, *p* = 0.0001, RMSEA = 0.12, *p*-value for test of close fit = 0.0001, CFI = 0.75, TLI = 0.70, and SRMR = 0.094. However, composite reliability for this one-factor construct was good (ρ_c_ = 0.84), indicating good reliability (values ≥0.6 is considered acceptable, while values ≥0.7 are good) [84, 90]. The alpha levels for the various measures indicated an acceptable inter-item consistency with Cronbach’s alpha coefficients of 0.65–0.90 (Table 1) and composite reliability of 0.80–0.84 (Table 3).

**Table 3:** Goodness-of-fit measures for OPQoL-brief measurement model. Confirmatory Factor Analysis for Model-1, Model-2 and Model-3

Fit Measure	Model-1 *N* = 181 13 items	Model-2 *N* = 181 9 items	Model-3 N = 181 8 items
χ^2^ *Satorra Bentler*	236.358	54.213	31.547
p-value	0.00001	0.001	0.048
$\frac{\chi^2}{df}$*Satorra Bentler*	3.33 (Df^1^ = 65)	2.008 (Df = 27)	1.58 (Df = 20)
RMSEA	0.121 (CI: 0.104–0.137)	0.074 (CI: 0.045–0.103	0.056(CI: 0.005–0.092)
*p-value (close fit test)*	0.000001	0.080	0.359
SRMR	0.094	0.060	0.050
CFI	0.76	0.93	0.97
TLI	0.70	0.91	0.95
Average Variance extracted (AVE)	0.300	0.324	0.340
$\mathrm{pc}=\frac{{\left(\Sigma \uplambda \right)}^2}{\left[{\left(\Sigma \uplambda \right)}^2+\Sigma \left(\uptheta \right)\right]}$	0.84	0.80	0.80

*OPQoL* Quality of Life measurement model. *RMSEA* Root Mean Square Error of Approximation. *SRMS* Standardized Root Mean Square Residual, *CFI* The Comparative Fit Index, *TLI* Tucker-Lewis Index, ^1^*Df* Degrees of freedom, *ρc* Composite reliability. Model-1: 13 items, Model-2: 9 items (items 6, 7, 10, 11 are dismissed). Model-3: 8 items (items 6, 7, 10, 11 and 12 are dismissed). Listwise N = 181

### Construct validity

An inspection of the standardized residuals and the modification indices (MIs), discovered five significant residuals [item7–3, (0.31) item6–4 (0.21), item5–6 (0.49), item 11–5(− 0.21), item11–6 (− 0.20)]. Furthermore, ten pair of items showed MIs higher than 10, all of which pointing to misspecifications. For the pairs of items 3–7 and items 5–6 the MIs were extremely high (MI = 15.10 and MI = 36.53, respectively).

Item3 (*‘I am healthy enough to get out and about’)* and item7 (*‘I am healthy enough to have my independence’)* contain physical functioning and thus share variance*.* Therefore, it is theoretically rational that they revealed a very high MI. Item3 loaded higher than item7; for that reason, item7 was dismissed from the model. The next step was to consider item6 (*‘I try to stay involved with things’),* which displayed an extremely high MI with item5 (*‘I have social or leisure activities/hobbies that I enjoy doing’)*. Item6 loaded significantly lower than item5 and was dismissed, and the model was run once more. This 11-items version gave somewhat better fit (χ^2^ = 137.62, (df = 44), χ^2^/df = 3.13, *p* = 0.0001, RMSEA = 0.11, *p*-value for test of close fit = 0.0001, CFI = 0.83, TLI = 0.78, SRMR = 0.080), although, a poor fit. Now, only one residual was significant, involving the pair of item11–5. Still, several very high MIs were found, involving item11 *(‘I take life as it comes and make the best of things’),* indicating this item to share unexplained variance with a number of other items. Hence, item 11 was excluded. Next, the estimates pointed at item10 (*‘I get pleasure from my home’*) and item9 (*‘I feel safe where I live’*); both concerned with resident’s sense of home while residing in a NH. Item9 showed the best loading and was kept, while item10 was set aside.

#### Model-2 – the OPQoL-brief 9-items unidimensional version

This modified version (including items 1,2,3,4,5,8,9,12,13), framed Model-2, gave an acceptable fit (χ^2^ = 54.21, (df = 27), χ^2^/df = 2.01, *p* = 0.001, RMSEA = 0.074, *p*-value for test of close fit = 0.080, CFI = 0.93, TLI = 0.91, SRMR = 0.06). However, even not a good fit.

#### Model-3 – the OPQoL-brief 8-items unidimensional version.

**Fig. 3:**
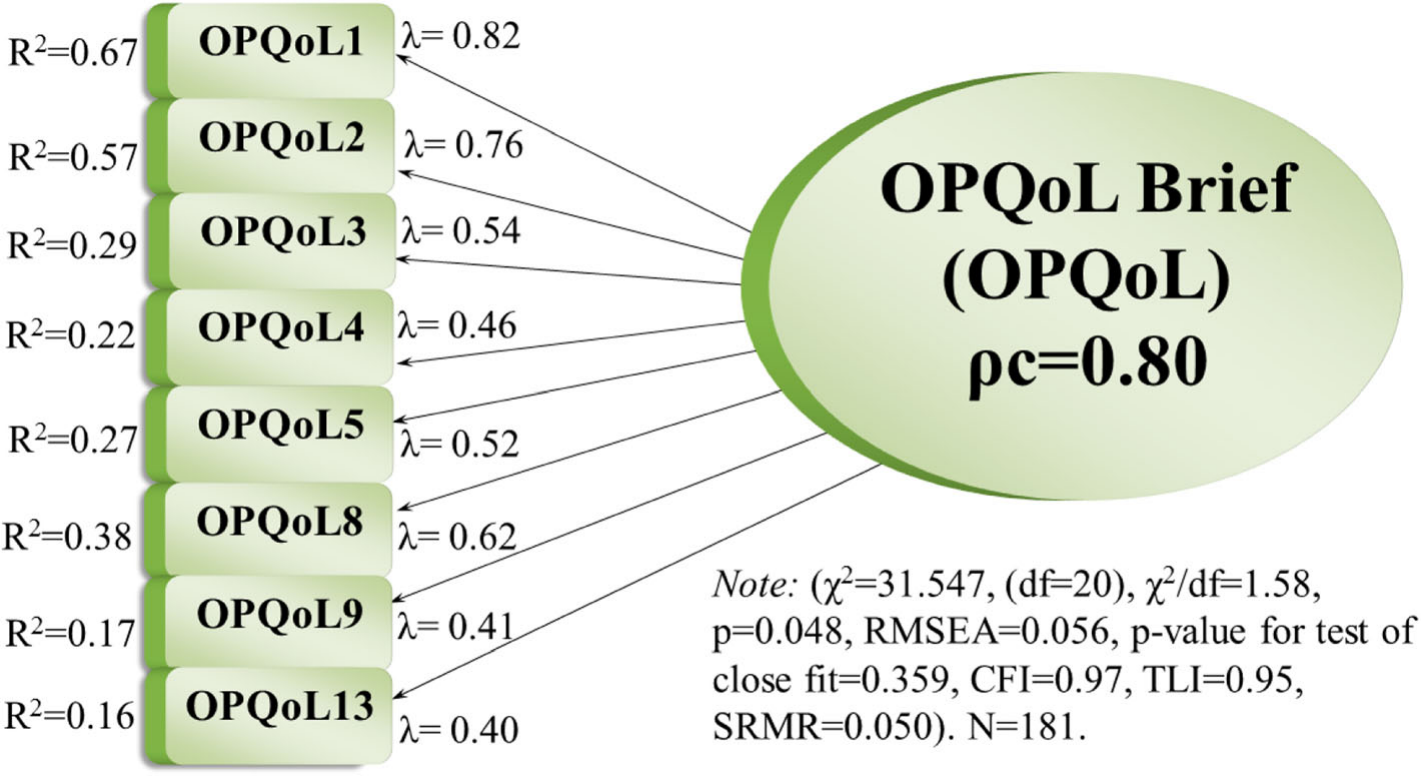
OPQoL-brief measurement model including 8 items (1,2,3,4,5,8,9,13). *N* = 181. Standardized factor loadings, multiple squared correlations, and composite reliability

Finally, dismissing item12 *(‘I feel lucky compared to most people’)* gave a god fit to the present data: χ^2^ = 31.55, (df = 20), χ^2^/df = 1.58, *p* = 0.048, RMSEA = 0.056, p-value for test of close fit = 0.359, CFI = 0.97, TLI = 0.95 and SRMR = 0.05. This version of the one-dimensional model including eight items (item 1,2,3,4,5,7,9 and 13) was framed Model-3, representing the best fitting model. Figure 3 portrays Model-3, showing the factor loadings, multiple squared correlations (R^2^), model fit and composite reliability (ρ_c_).

## Discussion

When evaluating a measurement scale, researchers face two important questions: (1) the underlying dimensionality of data (not too many, not too few factors), and (2) the adequacy of the individual items. This study assessed how well the original one-factor measurement model of the OPQoL-brief fit to the observed data, and whether the OPQoL-brief revealed good reliability and construct validity in a Norwegian NH population. Thus, the research question addressed evidence related to the dimensionality, reliability and construct validity of the OPQoL-brief questionnaire in this population.

### Dimensionality

The scree-test portrayed in Fig. 2 indicated that the number of factors to retain was three. However, two factors showed eigenvalues substantially higher than one, while the third factor was close to one (1.06), along with the next factors showing eigenvalues of 0.98, and 0.88, respectively. Hence, it seems not reasonable to regard the third factor with eigenvalue of 1.06 as ‘major’ and the fourth with eigenvalue of 0.98 as ‘trivial’. When it comes to determining the number of factors, Kaiser’s method (K1) sometimes is problematic and inefficient [91]. As seems to be the case here, the Kaiser-Guttman rule of retaining eigenvalues larger than 1 is not interpretively useful because it tends to result in the retention of too many factors [92]. Despite K1’s widespread use, experts agree that it has deficiencies and that its use is not recommended [92]. PCA needs to balance parsimony with adequately representing underlying correlations, so its utility depends on being able to differentiate major factors from minor ones [91]. By looking at the scree-plot for the PCA in the present study this issue seems evident; one strong factor along with several small factors were portrayed.

Moreover, the rotated 3-factor solution suggested by PCA revealed several cross-loadings with substantial factor loadings on all factors, thwarting the dimensionality. Only four (items 5,6,7,13) loaded solely on one dimension, indicating an unclear dimensionality of the construct and probably a one-dimensional solution like Bowling et al. [22] presented. Turning to CFA, the analyses suggested a unidimensional solution (Table 3). However, some items seemed troublesome, indicating misspecifications.

### Reliability

Reliability and construct validity are related to the adequacy of the individual items; highly significant standardized factor loadings–preferably > 0.7 indicates that the items perform as good indicators for the QoL construct in the NH population. The square of a standardized factor loading (R^2^), termed the variance extracted of the item, represents how much variation in an item the latent construct explains [93]. Loadings falling below 0.7 can still be significant, but more of the variance in the measure is error variance than explained variance. Looking at the factor loadings and the R^2^-values, only three items loaded good-excellent; item1 (λ = 0.82) was excellent, while item2 (λ = 0.69) and item8 (λ = 0.66) displayed good loadings. Contrasting this, item4 (λ = 0.41, R^2^ = 0.17), item6 (λ = 0.42, R^2^ = 0.18), and item13 (λ = 0.44, R^2^ = 0.19) performed like invalid indicators of QoL; the OPQoL-construct explained only a limited amount of the variance in these items. Consequently, the reliability of these indicators was low. The other seven items displayed fair factor loadings ranging between .46–.57. Hence, reliability was acceptable, but not fully supported. An examination of the inter-item correlations revealed plausible correlations (Table 5 in Appendix 2), with the highest values for the pair of items1–2 (r = .62), items5–6 (r = .55), items1–8 (r = .55) and items3–7 (r = .47). Moreover, Cronbach’s alpha (α) (Table 1) and composite reliability (ρ_c_) (Table 3) revealed good values, indicating good internal consistency [84, 90].

### Construct validity

Construct validity deals with the accuracy of measurement, reflecting the extent to which a set of measured indicators actually reflect the theoretical latent construct the items are designed to measure [94]. In the present study, convergent construct validity was supported by significant negative correlations between OPQoL-brief and HADS-A and HADS-D as well as positive correlations with ST1, ST2, PIL, NPIS and JOL (Table 1). Both hypotheses (H1 and H2) were supported. Items 1,2 and 8 revealed the best loadings, representing good indicators for QoL in the NH population. Interestingly, item8 (*‘I can please myself what I do’*) loaded strongly (.66), implying to be a valid indicator of QoL in this population. Considering that NH residents commonly experience idleness, spending many hours doing nothing, waiting, sleeping, this finding is noteworthy. Doing something, being active with something which you like, is essential for QoL among NH residents [95–99].

Content validity is a sub-form of construct validity, referring to whether the OPQoL-brief has an appropriate, relevant sample of items to represent the QoL construct. If the wording of items is too similar, a challenge occurs; items worded too similarly increase the average correlation among items, which in effect increases the coefficient alpha, yet without adding substantively to the content validity of the measure. Firstly, items 3 and 7 possibly are worded too closely; *‘I am healthy enough to get out and about’* (item3) and *‘I am healthy enough to have my independence’* seem to measure the same aspect. Staying in a NH without having dementia means that you on average have 6–7 diagnoses of chronic conditions [100], which negatively affect health, functioning and independency. Largely, cognitively intact NH residents are not healthy enough to get out and about. Due to illness and health problems, followed by care needs, they have moved to a NH. Consequently, their independency is impeded; many NH residents perceive their institutionalization as the beginning of their loss of independence and autonomy [101–103].

The notion that *‘I am healthy enough to have my independence…to get out and about*’ might not indicate independence in the NH life situation very well; in fact, it could be the opposite. Striving for independence while you are totally dependent on others might damage your QoL. Although some similarity among items of a scale is needed to tap into the domain, several items that are mere rewordings of other items are redundant and contain very little new information about the construct [40].

Secondly, the items concerning one’s home (items 9, 10), which for these older adults is a NH, might not be worded specific or precise enough. Many older adults in NHs do not experience the NH as their home [102], and are grieving over that they had to leave their home, representing a loss to them. The NH is the last stop in their life. The expression that *‘I get pleasure from my home’* (item10) might not be as central as it would be if these individuals were staying in their private home. However, *‘I feel safe where I live’* (item9) seems more appropriate; NH residents highlight the importance of feeling safe to their thriving and QoL [104, 105]. This population is characterized by high age, numerous losses, frailty, mortality, disability, powerlessness, dependency, vulnerability, poor general health, a high symptom burden and facing the end-of-life [19–21], all of which increases distress and vulnerability. Thus, feeling safe while staying in a NH seems closely connected to the nurse-patient relationship, care quality and nurse-patient interaction, more than being at ‘my home’. Hence, indicators including the NH working culture, milieu, atmosphere and nurse-patient interaction might be essential domains to include in a QoL measurement for NH residents [102]. Looking at the correlations between the summative scores (Table 1), QoL correlated highly with the nurse-patient-interaction, along with joy-of-life, interpersonal and intrapersonal self-transcendence and meaning-in-life. QoL correlated negatively with anxiety and depression. Thus, convergent construct validity was well supported.

Item12 (*‘I feel lucky compared to most people’*) did not explain a substantial amount of the variation in the OPQoL-construct (R^2^ = 0.24). It might be difficult to know who one should compare oneself with. If comparing with the healthy ones coping at home, one might not feel very lucky. Contrary, compared to those who are in hospital waiting for a place in a NH, one might feel lucky. Probably this indicator could be more specified towards the life situation of residing in a NH.

Finally, item11 (*‘I take life as it comes and make the best of things’*), revealed significant correlations with many items involved in the OPQoL-brief questionnaire. Possibly, item11 covers an attitude and coping mechanism which is very much needed and therefore commonly developed among NH residents. Consequently, this indicator largely relates with the other indicators, sharing variance, and thus blurring the dimensionality and the statistical fit. Including correlated error terms concerning item11 might be an option.

### Limitations

The shortened OPQoL-brief construct was supported by significant factor loadings, several goodness-of-fit indices and significant correlations in the expected directions with the selected constructs. However, a good model fit does not guarantee that we have obtained ‘the true model’; other alternative models might fit the data equally well as the model found [106].

The effective (listwise) sample size was *N* = 181, which is considered medium, and close to what is understood as a large sample size. A rate of 10 cases per observed variable is given as a rule of thumb [81, 90]. The models tested in this study included 13 items; accordingly, the sample of N = 181 should be enough. Out of 204 NH patients fulfilling the inclusion criteria, 188 participated, giving a response rate of 92%. This along with almost no missing data represent a strength of this study.

The OPQoL-brief scale was part of a questionnaire comprising 120 items. Accordingly, frail older NH residents might tire when completing the questionnaire, representing a possible bias to their reporting. To avoid such a bias, we carefully selected and trained experienced researchers in conducting the interviews following a standardized procedure, including taking small breaks at specific points during the process. This procedure worked out very well; all participants fulfilled the questionnaire without considerably difficulties. The fact that the researchers visited the participants in the NHs to help fill in the questionnaires might have introduced some bias on the respondents’ responses, which is a limitation of this study.

## Conclusion

This study suggests a unidimensional solution of the OPQoL-brief. However, five of the original 13 items emerged to be poor indicators of the OPQoL-construct showing fair reliability and an insufficient validity. The present study suggests that the nine- and eight-items versions revealed an acceptable and a good fit to the data, respectively. Further development and testing of a well-adapted scale assessing QoL in the NH population are required.

## Data Availability

The datasets generated and/or analysed during the current study are not publicly available due to Norwegian Act on medical and health research (ACT 2008–06-20 no. 44):§ 38 but are available from the corresponding author on reasonable request. All raw data is in Norwegian.

## Appendix 1

**Table 4:** The OPQoL-brief questionnaire. Original 13-items version, plus the preliminary single item on global QoL (OPQoL-G). Means and Standard deviation. During the last week, to what extent have you experienced that...

Variable	N	Mean	Std.Dev.
OPQoL1 I enjoy my life overall	181	3.63	1.082
OPQoL2 I look forward to things	181	3.89	0.958
OPQoL3 I am healthy enough to get out and about	181	3.48	1.269
OPQoL4 My family, friends or neighbours will help me if needed	181	4.27	1.014
OPQoL5 I have social or leisure activities/hobbies that I enjoy doing	181	2.99	1.251
OPQoL6 I try to stay involved with things	181	3.50	1.193
OPQoL7 I am healthy enough to have my independence	181	3.75	1.212
OPQoL8 I can please myself what I do	181	3.75	0.907
OPQoL9 I feel safe where I live	181	4.53	0.764
OPQoL10 I get pleasure from my home	181	4.01	1.000
OPQoL11 I take life as it comes and make the best of things	181	4.42	0.754
OPQoL12 I feel lucky compared to most people	181	4.07	0.864
OPQoL13 I have enough money to pay for household bills	181	4.42	0.835

Items 6, 7, 10, 11 and 12 are omitted in the best fitting 8-items measurement model

Listwise *N* = 181. The OPQoL-brief is scaled 1–5, where higher score means higher QoL

## Appendix 2

**Table 5:** Inter-item correlation matrix

OPQoL item	1 I enjoy my life overall	2 I look forward to things	3 I am healthy enough to get out and about	4 My family, friends or neighbors will help me if needed	5 I have social or leisure activities/hobbies that I enjoy doing	6 I try to stay involved with things	7 I am healthy enough to have my independence	8 I can please myself what I do	9 I feel safe where I live	10 I get pleasure from my home	11 I take life as it comes and make the best of things	12 I feel lucky compared to most people	13 I have enough money to pay for household bills
1 I enjoy my life overall	1,000												
2 I look forward to things	,622	1,000											
3 I am healthy enough to get out and about	,447	,399	1,000										
4 My family, friends or neighbors will help me if needed	,345	,409	,200	1,000									
5 I have social or leisure activities/hobbies that I enjoy doing	,395	,460	,226	,314	1,000								
6 I try to stay involved with things	,269	,422	,206	,348	,548	1,000							
7 I am healthy enough to have my independence	,281	,187	,465	,046	,224	,136	1,000						
8 I can please myself what I do	,547	,412	,353	,241	,363	,314	,341	1,000					
9 I feel safe where I live	,343	,300	,254	,228	,119	,030	,296	,235	1,000				
10 I get pleasure from my home	,349	,320	,271	,103	,239	,226	,355	,379	,481	1,000			
11 I take life as it comes and make the best of things	,426	,206	,304	,104	,025	-,017	,311	,383	,426	,310	1,000		
12 I feel lucky compared to most people	,357	,246	,216	,032	,220	,133	,285	,345	,304	,442	,375	1,000	
13 I have enough money to pay for household bills	,327	,281	,294	,253	,082	,126	,240	,224	,300	,209	,310	,320	1,000

## Abbreviations

CFI: Comparative Fit Index
HADS: Hospital Anxiety and Depression Scale
HADS-A: Hospital Anxiety and Depression Scale, subscale assessing anxiety
HADS-D: Hospital Anxiety and Depression scale, subscale assessing depression
JOL: Joy-of-life
JoLS: Joy-of-life scale
MSc: Master of Science
N: Sample size
NH: Nursing home
NPIS: Nurse-Patient-Interaction Scale
PIL: Purpose-in-life test
QoL: Quality of life
RMSEA: Root Mean Square Error of Approximation
RN: Registered nurse
SD: Standard Deviation
SEM: Structural Equation Modelling
SRMS: Standardized Root Mean Square Residual
ST: Self-Transcendence
ST1: Inter-personal Self-Transcendence
ST2: Intra-personal Self-Transcendence
STS: Self-Transcendence Scale
TLI: Tucker Lewis Index
WHO: The World Health Organization
